# Heat-Responsive Photosynthetic and Signaling Pathways in Plants: Insight from Proteomics

**DOI:** 10.3390/ijms18102191

**Published:** 2017-10-20

**Authors:** Xiaoli Wang, Chenxi Xu, Xiaofeng Cai, Quanhua Wang, Shaojun Dai

**Affiliations:** Shanghai Engineering Research Center of Plant Germplasm Resources, College of Life and Environmental Sciences, Shanghai Normal University, Shanghai 200234, China; wxl2006by@163.com (X.W.); chenxixu@shnu.edu.cn (C.X.); xfcai@shnu.edu.cn (X.C.); wangquanhuan@shnu.edu.cn (Q.W.)

**Keywords:** heat response, proteomics, photosynthesis, signaling

## Abstract

Heat stress is a major abiotic stress posing a serious threat to plants. Heat-responsive mechanisms in plants are complicated and fine-tuned. Heat signaling transduction and photosynthesis are highly sensitive. Therefore, a thorough understanding of the molecular mechanism in heat stressed-signaling transduction and photosynthesis is necessary to protect crop yield. Current high-throughput proteomics investigations provide more useful information for underlying heat-responsive signaling pathways and photosynthesis modulation in plants. Several signaling components, such as guanosine triphosphate (GTP)-binding protein, nucleoside diphosphate kinase, annexin, and brassinosteroid-insensitive I-kinase domain interacting protein 114, were proposed to be important in heat signaling transduction. Moreover, diverse protein patterns of photosynthetic proteins imply that the modulations of stomatal CO_2_ exchange, photosystem II, Calvin cycle, ATP synthesis, and chlorophyll biosynthesis are crucial for plant heat tolerance.

## 1. Introduction

Heat is one of the most severe environmental stress factors limiting crop growth and productivity [1,2,3]. Crop-based modeling and empirical studies in tropical and subtropical regions have reported 2.5–16% direct yield losses of major crops for every 1 °C increase in seasonal temperature [4]. Global warming leads to the continual increase of global mean temperature with 0.3 °C per decade [5], which has a serious threat to crop production. Photosynthesis contributes more than 90% of crop biomass, and thus enhancement of photosynthesis would increase crop yields [6]. However, photosynthesis is very sensitive to heat and easy to be inhibited under heat conditions. Alteration of any photosynthetic processes, such as photosystem II (PS II), PS I, electron transport chains, ATP synthesis, and carbon fixation always happened when plants were subjected to heat stress [6,7,8]. Therefore, improvement of photosynthesis efficiency is vital for crop production and could be a promising approach for increasing crop yield under heat stress.

Photosynthesis alteration in response to heat stress has been well reviewed in the past years [1,6,7,9,10,11,12]. Plant response to heat is a complex trait, dependent on heat intensity, duration time and plant species. Heat stress affects photosynthesis in the levels of physiological, biochemical and molecular aspects, such as destacking of thylakoid membrane, photosystem II damage, inhibition of cytochrome b6/f complex and ribulose-1, 5-bisphosphate carboxylase/oxygenase (RuBisCO), as well as reactive oxygen species (ROS) production. Accordingly, plants have developed several heat avoidant or tolerant strategies, such as stomatal closure, accumulation of osmoprotectants, modulation of fatty acids composition and saturation, increase of heat shock proteins, activation of ROS-scavenging enzyme activity, as well as synthesis of secondary metabolites [6]. Efforts have also been conducted to improve photosynthetic efficiency by engineering photosynthesis-related genes, such as *chloroplastic glutamine synthetase* (*GS2*), *chlorplastic Fe superoxide dismutase* (*FeSOD*), *ω-3 fatty acid desaturase* (*FAD7)*, *sedoheptulose-1,7-bisphosphatase* (*SBPase*), *C_4_-carboxylase phosphoenolpyruvate* (*C_4_-PEPC*), and transcription factors *ABA-responsive 17* (*ABR17*) [7,13].

Heat-responsive signaling is also crucial for the protection of photosynthesis apparatus under heat stress. Although the mechanism of plant heat sensing and signaling are still unclear, many signaling molecules (e.g., Ca^2+^ and ROS), hormones (e.g., ABA), protein kinases (e.g., calcium-dependent protein kinases (CDPK), and mitogen-activated protein kinase (MAPK)), and transcription factors (e.g., heat shock factor (HSF)) are known to participate in heat signal transduction. In addition, some candidate heat-responsive genes encoding heat shock protein 110 (HSP101), HSP90, HSF, abscisic aicd-responsive element-binding factor (AREB1) and WRKY11 have a high potential to be used for crop breeding with heat tolerance [13,14,15,16]. In spite of the intensive studies on plant heat tolerance, and the great progress by transgenic engineering and quantitative trait locus (QTL) molecular assisted breeding in recent years, a complete understanding of thermostolerant mechanism remains elusive. Moreover, there are still no economically practical technological means to improve photosynthetic capability and facilitate crop production under heat stress.

Because of the posttranscriptional and posttranslational modifications, the changes of protein abundance can provide direct understanding of heat adaptive mechanism. Two-dimensional electrophoretic (2DE) and two-dimensional fluorescence difference in gel (DIGE) approaches have been widely used for separating heat-responsive proteins in plant. 2DE is a powerful technology with high resolution in protein separation, especially for protein isoform analysis, and DIGE overcomes some of the limitations of 2DE (e.g., throughput, reproducibility and sensitivity) [17]. The differentially expressed protein spots on 2DE/DIGE can be identified by matrix-assisted laser desorption/ionization tandem time-of-flight mass (MALDI-TOF/TOF MS) and liquid chromatography electrospray ionization (ESI) MS system (LC-MS) [18]. Importantly, a gel-free quantitative proteomics strategy, isobaric tags for relative and absolute quantitation (iTRAQ), has been applied for proteomic quantitation with a high throughput, sensitivity and efficiency, especially for identifying low abundant proteins (e.g., transmembrane receptors, intracellular kinases, and transcription factors) [18]. In previous studies, 2DE/DIGE-based proteomics was the main approach popularly employed in investigation of plant heat-responsive proteins (Table 1). Among them, more than 500 heat-responsive photosynthetic and signaling protein species have been identified in leaves from 16 plant species, including eight dicots (i.e., *Brassica oleracea*, *Carissa spinarum*, *Glycine max*, *Medicago sativa*, *Portulaca oleracea*, *Raphanus sativus*, *Arabidopsis thaliana*, and *Vitis vinifera*) and eight monocots (i.e., *Agave americana*, *Agrostis* sp., *Hordeum* sp., *Miscanthus sinensis*, *Oryza* sp., *Pinellia ternata*, *Zea mays* and *Triticum aestivum*) (Table 1 and Table S1). Here, we review the critical heat-responsive signaling and photosynthesis mechanisms in leaves revealed from these proteomic publications.

## 2. Heat-Responsive Signaling Transduction

The heat signal perception and transduction are very important steps for plant stress tolerance. At least four sensors are proposed to trigger heat stress response, including a plasma membrane channel that initiates an inward calcium flux, a histone sensor in the nucleus, and two unfolded protein sensors in the endoplasmic reticulum and the cytosol [52]. The downstream heat-responsive signaling pathways are predicted to be Ca^2+^ signaling, G protein-mediated signaling, and kinase signaling. Recent proteomic studies found several signaling proteins, which provided new clues for understanding the complex heat stress signal transduction (Table 2).

### 2.1. Ca^2+^ Signaling Pathways

It is well known that the inward flux of calcium serves as one of the primary heat sensors of plants [52], and regulates multiple signaling pathways in plants in response to heat stress [53]. In proteomics results, the abundances of calcium-binding protein and a Ca^2+^-transporting ATPase-like protein were increased in chloroplasts from *A. americana* leaves under 55 °C heat treatment [19]. Calcium-binding proteins participate in calcium cell signaling pathways by binding to Ca^2+^, which are involved in the activation of multiple kinases and transcription factors in heat stress. Importantly, a calmodulin-binding family protein showed a significant increase of phosphorylation level [51] activating Ca^2+^-calmodulin-dependent processes in response to heat stress [54]. In contrast, an isoform of annexin in *A. thaliana* leaves was declined in response to heat stress [22]. Annexin is a conserved family of eukaryotic Ca^2+^-dependent phospholipid-binding protein, which participates in the response of environmental stress [55,56]. *A. thaliana* annexin 1 mediates a plasma membrane calcium-permeable conductance in roots activated by ROS. An *O. sativa* annexin interacted with MAPK and was involved in Ca^2+^-dependent MAPK signaling pathway [55,57]. However, the role of annexin in heat signaling is still unclear.

### 2.2. G Protein-Mediated Signaling

Proteomics also found several G proteins showed increased trends in leaves in response to heat stress, such as an isoform of G protein in *P. oleracea* [42], a Rab1C in *G. max* [26] and four isoforms of Ran1A in *Agrostis* sp. [20]. It is well known that G proteins constitute one of the most important cell signaling cascades and participate in multiple signaling pathways [58,59]. Previous studies have shown that some isoforms of G proteins (e.g., G alpha, G beta, G gamma, and Rab7) are associated with plant heat tolerance [60,61,62,63]. These findings indicate the possible function of these G proteins/small G proteins in heat sensing, thus bringing novel perspectives in understanding the complex heat stress signal transduction.

### 2.3. Kinase Signaling Pathways

The heat signals transmitted by second messengers activate multiple protein kinases (e.g., CDPK and MAPK). Current proteomic studies found the increased abundances of nucleoside diphosphate kinases (NDPKs) in heat-stressed leaves of *G. max* [26] and *O. sativa* [34]. NDPK can interact with H_2_O_2_-mediated mitogen-activated protein kinase (MAPK) signaling and associate with plant heat tolerance [64]. The increase of NDPK has also been reported in plants to cope with other environmental stresses, such as drought, salt and cold [34,65]. We therefore propose that the accumulation of NDPK is important for plant stress tolerance. Similarly, a BRI1-KD interacting protein 114, which contains NDPK group I like domain, was increased in heat-tolerant *T. aestivum* cultivar, but did not change in heat-sensitive cultivar [49]. Previous studies have shown that the increased BRI1-KD interacting protein 114 play roles in alleviating effects of salt stress in a tolerant *T. aestivum* cultivar [66]. Their detailed roles in plant heat signaling still need to be further investigated.

In addition, the phosphorylation levels of two protein kinases (i.e., serine threonine-protein kinase wnk4-like and protein kinase superfamily protein) and phospholipase C (PLC) were also changed in response to heat stress [51]. It is well known that serine/threonine (Ser/Thr) phosphorylation plays key roles in the regulation of plant stress response. PLC is a major membrane phospholipid hydrolyzing enzyme, and its phosphorylated form is involved in the regulation of various cellular processes in plants in response to stress conditions, such as heat, salt and drought [67,68,69,70].

### 2.4. Heat-Responsive Transcription Factors

Proteomic studies also found several transcription factors involved in plant heat signal transduction, such as WRKY and MYB. Both of them were increased in *P. oleracea* leaves upon 35 °C treatment [42]. *P. oleracea* is a typical C_4_ and thermotolerant plant species widely distributed in tropical regions. The increase of WRKY and MYB in response to heat stress may be correlated with the great heat tolerance of *P. oleracea*. MYB proteins are involved in regulating signal transduction and biosynthesis of secondary metabolites [71]. Overexpression of *OsMYB55* improves *O. sativa* tolerance to heat by enhancing amino acid metabolism through transcription activation [72]. WRKY acts as key component in abscisic acid signaling and play roles in multiple stress tolerances [71,73]. *OsWRKY11*-overexpressing *O. sativa* exhibited enhanced tolerance to drought and heat [74]. All these indicate that MYB proteins and WRKY can act as very promising targets to improve crop heat stress tolerance.

**Figure 1:**
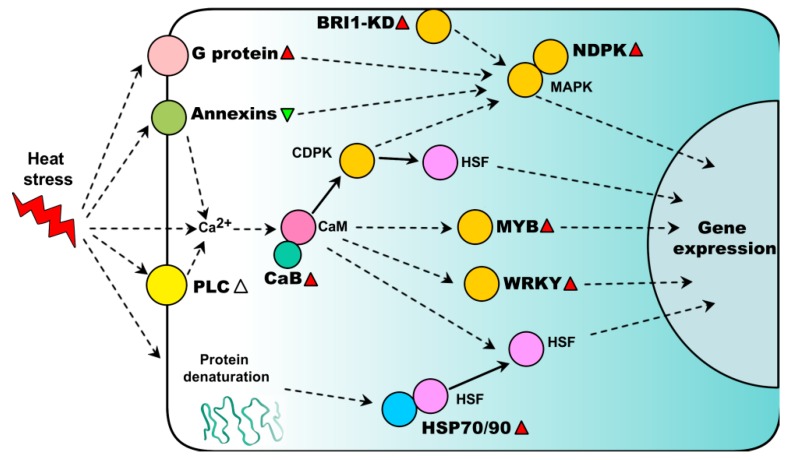
Schematic representation of heat-responsive proteins involved in signal transduction pathway. Heat stress results in an inward flux of calcium and activation of G protein by phospholipase C (PLC), annexins and other proteins. Calcium binds the calmodulin (CaM) and calcium-binding protein (CaB), and then activates multiple kinases (e.g., CDPK and MAPK) and transcriptional regulators (e.g., MYB, WRKY and HSF). HSP70 and HSP90 negatively regulate the activity of HSFA1. BRI1-KD and annexin participate in the heat signal transduction through MAPK-activated stress response. Red and green triangles represent heat-increased and heat-decreased abundance of proteins, respectively. White triangle represents heat-increased phosphorylation level. Partly adopted from Mittler et al. [52].

Additionally, the activity of heat shock transcription factor A1 (HsfA1) was negatively regulated by HSP 70 and HSP90 [75]. Proteomics studies reported that several HSPs (e.g., HSP70s, HSP90s, and small HSPs) were induced in many plant species under heat treatments [22,26,41,45] (Table S1). HSPs are well known for their critical role in maintaining protein folding under heat stress. A schematic model of the heat signaling pathway mediated by novel signaling components discovered in proteomic studies is shown in Figure 1.

## 3. Chlorophyll Synthesis Is Disturbed by Heat Stress

Chloroplasts are the major organelles for photosynthesis, and chlorophyll (Chl) is the most important pigment for light absorbance during photosynthesis. It has been reported that Chl biosynthesis is inhibited when plants were exposed to heat stress, which is probably due to the heat destruction of several enzymes in Chl biosynthesis [76,77], such as protochlorophyllide reductase (POR) and magnesium-chelatase subunit. POR is responsible for the photoreduction of protochlorophyllide to chlorophyllide. Previous study indicated that the abundance of POR was decreased in heat-stressed *T. aestivum* seedlings associated with the reduced accumulation of Chl [78]. Moreover, in heat-stressed *Z. mays*, the phosphorylation level of POR was decreased by heat stress [51] (Table S2). It has been suggested that POR phosphorylation can facilitate the aggregation of protochlorophyllide and POR, while the aggregation is needed for the next catalytic reaction for POR [79]. These suggested that not only the abundance but also the phosphorylation level of POR may contribute to the Chl biosynthesis. Magnesium-chelatase catalyzes the insertion of magnesium ion into protoporphyrin IX to yield Mg-protoporphyrin IX. A magnesium-chelatase subunit ChlI-2 identified in *V. vinifera* leaves were repressed after heat stress [50]. However, an isoform of magnesium-chelatase subunit of POR was significantly increased in leaves of *P. oleracea* under 35 °C heat stresses for 6–24 h [42]. The increased abundance of magnesium-chelatase subunit may be related to the excellent photosynthetic capability of *P. oleracea* under heat stress. Further functional analyses of magnesium-chelatase subunit and chlorophyll-binding proteins in heat-treated *P. oleracea* leaves are needed.

## 4. Photosystem (PS) II and PS I Are Disrupted under Heat Stress

**Figure 2:**
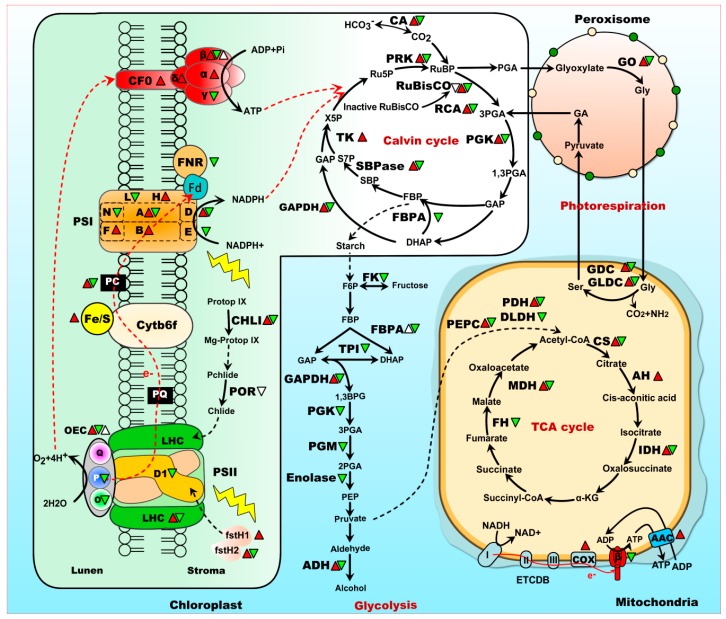
Schematic representation of heat-responsive photosynthesis and carbon metabolism revealed from proteomics. Red triangle and green triangle represent heat-increased protein abundance and heat-decreased protein abundance, respectively. White regular triangle and white inverted triangles represent heat-increased phosphorylation level and heat-decreased phosphorylation level, respectively. The solid line indicates single-step reactions, and the dashed line indicates multi-step reactions. ADH, alcohol dehydrogenase; AH, aconitate hydratase; AMY, β-amylase; CA, carbonic anhydrase; CHLI, magnesium-chelatase subunit; COX, cytochrome c oxidase assembly protein; Cytb6f, cytochrome b6/f complex; D1, photosystem II reaction-center D1 protein; DLDH, dihydrolipoyl dehydrogenase; FBPA, fructose-bisphosphate aldolase; Fe/S, Rieske Fe/S protein of cytochrome b6/f complex; FK, fructokinase; FNR, ferredoxin—NADP reductase; FPK, fructose-6-phosphate-2-kinase; fstH, filamentation temperature-sensitive H; GAPDH, glyceraldehyde-3-phosphate dehydrogenase; GDC, glycine decarboxylase; GLDC, glycine dehydrogenase; GO, glycolate oxidase; IDH, isocitrate dehydrogenase; LHC, chlorophyll a/b binding protein; MDH, malate dehydrogenase; NDUFV, NADH2 dehydrogenase; OEC, oxygen-evolving enhancer protein; PDH, pyruvate dehydrogenase; PEP, phosphoenolpyruvate carboxylase; PGK, phosphoglycerate kinase; PGM, phosphoglycerate mutase; PK, pyruvate kinase; PRK, phosphoribulokinase; PSI, photosystem I; PSII, photosystem II; RCA, ribulose-1,5-bisphosphate carboxylase/oxygenase activase; Rubisco, ribulose-1,5-bisphosphate carboxylase/oxygenase; SBPase, sedoheptulose-1,7-bisphosphatase; SHMT, serine hydroxymethyltransferase; SUS, sucrose synthase; TPI, triosephosphate isomerase; UPGase, UDP-glucose pyrophosphorylase.

PSII is one of the most thermosensitive components of photosynthetic apparatus [80,81]. The water oxidizing complex (WOC), PSII reaction center, and light harvesting complexes are all initially disrupted under heat stress. Some of the heat-responsive proteins involved in PSII are summarized in Figure 2. Among them, chlorophyll-binding proteins are involved in harvesting light energy and transferring it to photochemical reaction centers. As revealed by proteomics studies, chlorophyll-binding proteins were generally decreased in heat-treated *H. vulgare* [29] and *V. vinifera* [50] ((Table 2 and Table S1). In the chloroplasts of leaves from *A. americana*, the protein abundance and transcript of light-harvesting chlorophyll a/b binding protein (LHC) were all decreased under heat stress [19]. However, chlorophyll-binding protein species in leaves of *P. ternata* exhibited a substantial increase in response to 38 °C heat stress for 24 h [41]. In heat stressed leaves of *M. sativa*, three chlorophyll a/b binding proteins were increased at 24 h, but decreased in the prolonged heat stress course (72 h) [31]. These results indicate that enhanced abundances of chlorophyll-binding proteins may be somehow related to heat tolerance, depending on the stress intensity, duration, and plant species.

Heat inactivation of PSII is mainly due to the dissociation of oxygen-evolving complex (OEC) [6]. OEC is involved in the photo-oxidation of water during the light reactions of photosynthesis. It is more sensitive and easy to be inhibited than the reaction center of PSII, when plants were exposed to heat stress even for a short time. In proteomics studies, the two most important members of OEC, oxygen-evolving enhancer protein 1 (OEC1) and OEC2, normally showed a decrease in some plant species, such as *G. max* [26], *A. americana* [19], *M. sinensis* [32], *O. sativa* [35], *Agrostis* sp. [21], *R. sativus* [44], and *C. spinarum* [25], which was supposed to be the main reason of the inhibition of photosynthesis. Additionally, two OEC subunits, PsbP and PsbO, also significantly decreased in *O. sativa* when exposed to heat (e.g., 35 °C, 40 °C, and 45 °C) treatment for 48 h [35], which may contribute to the decreased abundance of OEC [80,82,83]. In addition, an isoform of OEC1 with significantly heat-induced phosphorylation level at Ser-3 site was found in leaves of *Z. mays* [51]. Previous studies suggest that phosphorylation partially suppressed the release of three OEC subunits, PsbO, PsbP and PsbQ (17KD), from PSII membranes under light stress [84]. Thus, we suppose that the increased OEC1 phosphorylation would alleviate the damage of OEC1, but its regulatory mechanism remains to be investigated.

To coping with heat stress, plants have developed a sophisticated mechanism to repair PSII damage [10]. PSII reaction-center D1 protein turnover plays a key role in the PSII repair cycle. Chloroplastic degradation (Deg) proteases, filamentation temperature-sensitive H (fstH) proteases and other proteases participate in the proteolysis of D1 protein [85,86]. Proteomics studies found an increased abundance of cysteine proteinase in leaves of *A. americana* [19], and fstH1 protease in *Hordenm spontaneu* [29] and *Hordenm vulgare* [30] when exposed to heat stress (Figure 2 and Table S1). Moreover, fstH2 was increased in heat-tolerant *T. aestivum* cultivar but decreased in heat-sensitive one [49], implying fstH2 would be an interesting target for crop breeding. In *A. thaliana*, fstH11 has been suggested to have a direct role in thermotolerance, which can protect the photosynthesis apparatus from heat stress [87].

It has been reported that PSI activity is much more heat stable than PSII. However, several PSI subunits (e.g., PsaA, PsaD, PsaE, and PsaN) were also generally heat-decreased in *A. americana*, *A. thaliana*, *A. stolonifera*, and *A. scabra*, respectively [19,21,22](Figure 2). The abundances of PsaB were increased in 55 °C heat-treated leaf chloroplasts of *A. americana*, but its transcript level was induced at 45 °C then suddenly decreased at 55 °C and 65 °C [19]. PsaA and PsaB are both involved in binding electron transport cofactors P700. PsaD and PsaE provide the docking sites for soluble electron transporter ferredoxin on the stromal side of the thylakoid membrane. Their decrease in abundance implies that the energy transfer in PSI is inhibited under heat stress. However, because PSI activity is difficult to assay [88], the detailed function of these proteins in PSI in response to heat stress is still unknown.

## 5. CO_2_ Fixation Is Inhibited Under Heat Stress

Heat-inhibition of photosynthetic CO_2_ fixation has been documented in many studies. The heat reduction of CO_2_ fixation is due to low production of ATP and NADPH from the light reactions, as well as decreased intercellular CO_2_ concentration under heat stress. Both of these in turn significantly affect the activities of key photosynthetic enzymes, such as carbonic anhydrase (CA), RuBisCO, RuBisCO activase (RCA), and phosphoribulokinase.

CA is a zinc-containing metalloenzyme, which catalyzes the reversible hydration of CO_2_ and functions in CO_2_ exchange by influencing the internal stomatal conductance [89]. Under heat stress, five isoforms of CA were decreased in *G. max* cv Enrei under 40 °C for 12 h [26]. However, CAs was heat-increased in heat-tolerant *G. max* variety, but decreased in heat-sensitive one when expose to 42 °C heat stress for six days [27]. In addition, four isoforms of CA in *Agrostis* sp. were heat-increased [21]. These suggest that CAs may play important roles in various plant species/cultivars with different tolerance abilities. Moreover, functional analyses have shown that overexpressing *O. sativa* CA gene significantly improved the heat tolerance of *E*. *coli* recombinant [90], which reinforces the functional evidence for the potential role of CA in heat tolerance.

Proteomic studies revealed that some isoforms of the RuBisCO large subunits and small subunits were decreased in several plants under heat stress [25,26,49] (Table 2 and Table S1). Moreover, heat stress also induced dephosphorylation of RuBisCO [40], which is suspected to decline RuBisCO activity [68]. RuBisCO activity is regulated by RCA. RCA functions as maintenance and acclimatization of photosynthetic CO_2_ fixation, increases the photosynthetic rate during heat stress [31]. RCA is easy to be dissociated by heat stress, which causes a reduction in the photosynthetic capacity [91]. Proteomic studies revealed the abundances of RCAs in *V. vinifera* and *Agrostis* sp. were significant decreased [21,50], which in is in accordance with the inhibition of the activities of RCAs [92,93]. Interestingly, several isoforms of RCA were heat-increased in *O. sativa* [35], *C. spinarum* [25], and *T. aestivum* [45,49]. Most plants have two forms (α- and β-isoform) of RCA which results from alternative splicing of one RCA pre-mRNA. Both α- and β-isoform are capable of promoting RuBisCO activation but they differ markedly in their enzyme activity and sensitivity to thermal denaturation [94,95]. For instance, the heat-induced RCA α-isoform in *O. sativa* plays an important role in photosynthetic acclimation to moderate heat stress, while RCA small isoform (β-isoform) plays a major role in maintaining RuBisCO initial activity under normal conditions [94].

Moreover, several enzymes involved in the regeneration of ribulose bisphosphate (RuBP) were widely decreased under heat stress, such as phosphoribulokinase (PRK) in *O. sativa*, *M. sinensis*, *A. stolonifera*, and *A. scabra*, glyceraldehyde 3-phosphate dehydrogenase (GAPDH) A/B subunits in *A. stolonifera* and *A. scabra*, fructose-bisphosphate aldolase (FBPA) in *G. max* and *M. sinensis*, as well as sedoheptulose-1,7-bisphosphatase (SBPase) in *G. max* and *M. sinensis* [26,32,34,49]. These enzymes play key roles in carbon flux in Calvin cycle, determining carbon assimilation and photosynthesis rate [96]. The heat-decreased abundances of these enzymes imply carbon fixation or assimilation is highly disturbed in plants under heat stress. Among these enzymes, SBPase is a major control point in the C_3_ cycle [91], and it is possible to increase photosynthesis carbon fixation by increasing the level of SBPase under heat stress conditions. The decrease of SBPase in transgenic tobacco resulted in the decrease of photosynthetic capacity [97], while overexpression of *SBPase* enhanced the photosynthesis in transgenic *O. sativa* plants under heat stress [98]. An isoform of SBPase in heat-tolerant plant *O. meridionalis* consistently increased in protein abundance, while its gene expression was declined under heat stress [33], indicating SBPase may be a transient response to protect the photosynthetic machinery during heat stress. The similar observation also found in another two photosynthesis-related enzymes (i.e., phosphoglycerate kinase and PRK) in *O. meridionalis* [33], which indicated that the heat-responsive function was regulated at both transcript and translational levels. Besides, GAPDH is a key enzyme for the conversion of glycerate-3-phosphate to glyceraldehyde-3-phosphate interacting with ATP and NADPH, where glycerate-3-phosphate can accept electrons from NADPH, preventing the ROS-induced deceleration of PSII repair. In proteomics studies, a heat-increased chloroplastic GAPDH A subunit was found in tolerant *T. aestivum* cultivar under 35 °C/26 °C (day/night) heat treatment for five days, which was in contrast to that in sensitive *T. aestivum* cultivar [49]. In *A. thaliana*, overexpressing *ThGAPB* exhibited higher recycling rates of ADP and NADP^+^, which helps to maintain photosynthetic efficiency by reducing ROS production under stress conditions [99]. In addition, several isoforms of chloroplast transketolase (TK) in *O. meridionalis* and *O. sativa* were generally increased in heat-stressed leaves, which would facilitate to recover the activity of RuBisCO to cope with heat stress [34].

Additionally, C_4_-specific pyruvate orthophosphate dikinase (C_4_-PPDK) was found increased during heat stress and heat recovery processes in leaves of C_4_ *M. sinensis* [32]. C_4_-PPDK catalyzes the formation of phosphoenol pyruvate, the initial acceptor of CO_2_ in the C_4_ photosynthetic pathway. The increased abundance of C_4_-PPDK plays an important role in enhancing the photosynthesis of C_4_ plant to cope with heat stress [100]. In heat stressed *Z. mays*, the phosphorylation level of phosphoenolpyruvate carboxykinase (PEPCK) was decreased, whereas its protein abundance did not change [51]. The PEPCK involved in C_4_ photosynthesis catalyzes the release of CO_2_ from oxaloacetate for Calvin cycle. The phosphorylation of PEPCK may contribute to the coordinate regulation of the activity of PEPCK [101,102], which performs notably the initial fixation of CO_2_ in the photosynthetic carbon metabolism of C_4_ plants. Transferring the C_4_ enzymes to C_3_ plants is often thought as a useful strategy to improve the photosynthetic performance of C_3_ plants. Some C_4_ enzymes encoding genes, such as *C*_4_*-PPDK* [103], *C*_4_*-PEPC* [104], and *C*_4_*-SBPase* [105], have been conducted on generating transgenic plant to improving C_3_ photosynthetic performance under abiotic stresses. However, now more and more researchers found that transferring single C_4_ enzyme to improve photosynthetic capacity is limited. Generating transgenic plant with multiple C_4_ genes or other photosynthesis-related genes should be considered for enhance of plant tolerance.

Heat stress not only affects carbon fixation, but also adjusts respiratory carbon metabolism in leaves. Among the 74 heat-responsive proteins involved in various carbon metabolism pathways (e.g., glycolysis, pentose phosphate pathway and TCA cycle) from ten plant species, 56 protein species were heat-decreased, while 18 protein species were heat-increased (Table 2 and Table S1). This implies that respiratory carbon metabolism is significantly inhibited in plants under heat stress. However, the heat response of glycolysis and TCA cycle were various in different plant species. For example, most proteins were decreased in *Agrostis* sp., implying carbon metabolism was heat-inhibited. GAPDHs were generally decreased in heat-sensitive cultivars, but increased in heat-tolerant ones. In addition, four enzymes (i.e., GAPDH, malate dehydrogenase, pyruvate dehydrogenase and transketolase) were generally heat-increased in leaves of *O. sativa* [34,35], but four enzymes in TCA cycle (i.e., dihydrolipoyl dehydrogenase, aconitate hydratase, malate dehydrogenase, and citrate synthase) were heat-decreased in *V. vinifera* leaves [48]. This is consistent with what was found in transcriptome analysis, i.e., that the glycolysis-related genes (e.g., *phosphofructokinase* and *pyruvate decarboxylase*) were up-regulated in rice leaves, but most TCA cycle-related genes were down-regulated [106].

## 6. Photorespiration Is Enhanced to Cope with Heat Stress

Photorespiration can protect photosynthesis from photoinhibition and prevent ROS accumulation in green tissues [82], and is believed to play various roles in plants stress resistance amino acid metabolism and signal transduction. In proteomics results, a key photorespiration-related protein, glycolate oxidase (GO), catalyzing the oxidation of glycolate to glyoxylate, was increased in *P. oleracea* [42] but decreased in leaves of *V. vinifera* [50] under heat stress. It is known that *P. oleracea* is a typical C_4_ plant which has higher heat tolerance and excellent photosynthetic capability, wherase *V. vinifera* is a C_3_ plant. The different GO abundances under heat stress between the two species may be related to their different photosynthetic capability. Previous studies have shown that rice photosynthesis and growth was positively correlated with GO activity [107]. In *O. sativa*, overexpression of *GO* significantly improved photosynthesis under high light and heat stress [107], whereas, suppression of *GO* inhibited photosynthesis through deactivating RuBisCO in rice [108]. All these results imply that increase of GO facilitates maintaining photosynthetic activity, which could be taken as a candidate gene for improving plant photosynthesis.

In addition, glycine decarboxylase (GDC) in *T. aestivum* [49] and glycine dehydrogenases (GLDC) in *O. sativa* [35] and *O. meridionalis* [33] were also heat-responsive (Figure 2). GDC is important in maintaining electron flow in order to prevent photoinhibition under stress conditions. As revealed by proteomics studies, GDC was increased in heat-tolerant genotype of *T. aestivum*, but decreased in heat-sensitive genotypes [49]. Similarly, GLDCs were increased in leaves of heat tolerant *O. meridionalis*, but decreased in rice. This suggests that proteins involved in photorespiration were more stable in heat-tolerant cultivars/species, which could be necessary for leaves to cope with heat stress. The glycine cleavage system H protein in rice leaves was also increased [35]. Overexpression of *glycine decarboxylase H* could considerably enhance the net-photosynthesis and growth of *A. thaliana* [109]. Besides, chloroplastic glutamine synthetase precursor was significantly increased in rice seedlings under 40 °C and 45 °C treatments. Chloroplastic glutamine synthetase is also a key enzyme involved in photorespiration, which catalyzes the ATP-dependent condensation of ammonia with glutamate to yield glutamine. The increases of these photorespiration-related proteins indicated that plant was partly protected from the photo-oxidation damage when exposed to heat stress.

## 7. Energy Supply Is Essential for Heat Tolerance

As revealed by proteomics studies, some heat-responsive proteins involved in energy metabolism were altered by heat stress (Table 2 and Table S1). The abundances of α, β, γ, and δ subunits of ATP synthase were heat-changed in plant species/genotypes (Figure 2). For example, ATP synthase γ subunit showed significantly decrease in leaves of *Agrostis* sp. and *A. thaliana*, while ATP synthase α subunit (in *T. aestivum*) and β subunit (in *T. aestivum* and *B. oleracea*) were increased in heat-tolerant plant genotypes but decreased in heat-sensitive genotypes [24,49]. The ATP synthase γ subunit is believed to be important in regulating ATP synthase activity and the flow of protons through the CF_0_ complex. The decreased ATP synthase γ subunit indicated the reduction of ATP synthesis in these plants under heat stress. The changes in abundances of these ATP synthase subunits exposed to heat stress indicated that ATP synthase is important in maintaining the energy metabolism under heat stress conditions. In heat-stressed rice leaves, ATP synthase activity were reduced, along with an decrease abundance of ATP synthase β subunit but an increased abundance of α subunit [34]. Moreover, an increased phosphorylation level of ATPase β subunit was found in heat-stressed rice leaves [40]. It has been reported that phosphorylation reduced the activity of ATP synthase [110]. The CF1 α subunit is the largest subunit of the ATP synthase, which displays an organizing function in the assembly of the multi-subunits of ATP synthase upon thermal denaturation [50]. Interestingly, all the identified ATP synthase subunits (i.e., β, γ, and δ subunit of CF_0_) in heat-stressed *V. vinifera* were induced under heat stress, and all of them recovered to their control levels after subsequent recovery [50]. The increase of these subunits may be a strategy for *V. vinifera* to adaption and recovery from heat stress. Besides, the abundance of ATP synthase δ subunit was significantly increased in heat-tolerant *C. spinarum* [25]. All these indicated that maintenance of ATPase abundance and activity is crucial for energy supply in heat-tolerant genotypes.

The electron transport in photosynthetic process was also significantly affected by heat stress. Ferredoxin-NADP(H) oxidoreductase (FNR), a key enzyme in photosynthetic electron transport, which catalyzes the last enzymatic step of the noncyclic photosynthetic light reaction responsible for the reduction of NADP^+^ in the PSI complex, were found decreased in heat-stressed leave of *G. max* [26] and *V. vinifera* [50]. In addition, FNR exhibited both heat-reduced abundance and gene expression in leaves of *O. meridionalis* [33], implying photosynthetic electron transport was highly inhibited by heat stress. Another photosynthetic electron transport related protein, plastocyanin, was increased in leaves of *M. sativa* under short-term (24 h and 48 h) heat stress, and then decreased under long-term (72 h) heat stress [31]. However, Rieske Fe/S protein of cytochrome b6/f complex was heat-increased in leaves of *P. ternata* [41]. The Rieske Fe/S protein of the cytochrome *b6/f* complex is an indispensable component of photosynthetic electron transport chain in chloroplasts [111]. All these indicate that heat tolerance in plants is a cost-intensive process and needs considerable cellular energy to cope with adversaries of heat [112].

## 8. Conclusions and Perspectives

Heat-responsive signaling and photosynthetic modulation are fine-tuned and critical pathways for plant tolerance. Previous transcriptomics and molecular genetics studies have identified and characterized some heat-responsive genes underlying the signaling and photosynthetic mechanisms. Current proteomics provided more protein abundance information for interpreting the alteration of these pathways, such as heat signal transduction, PSII repair, electron transport, PSI activity, CO_2_ fixation, ATP synthesis, and photorespiration. Moreover, the heat-responsive phosphorylation levels of some important proteins (e.g., POR, OEC, RuBisCO and PEPCK) implied that post translational modification (PTM) is crucial during the processes of plant heat tolerance. Importantly, some representative heat-responsive proteins (e.g., HSP, ftsH11, MYB, WRKY, and SBPase) have potential to improve heat stress tolerance in plants, although most of the heat-responsive proteins identified from proteomics approaches still need to be characterized by molecular genetics. In addition, most of the protein information is generated from 2DE gel-based proteomics approach, the throughput and sensitivity of which are much lower than those of gel-free approaches such as iTRAQ or tandem mass tag (TMT) label and label-free methods. The limitation of protein separation and label method probably resulted in most low abundant proteins, such as signal molecules, transcription factors, thylakoid membrane protein, and kinase, were missed in identification. Therefore, the high-throughput labeling proteomic and PTM analysis (e.g., S-nitrosylation, glycosylation and ubiquitination) would facilitate by providing more information for heat-response signaling and photosynthetic networks in plants [113].

## Figures and Tables

**Table 1:** Summary of current publications on heat-responsive proteomics.

Plant Species	Tissue/Organ	Variety	Treatment Conditions	Platforms	Protein Species ^(^^a)^	Unique Proteins ^(b)^	Reference
*Agave americana*	leaf chloroplast	nd	55 °C; 4 h	2DE, ESI-Q-Trap MS	58	58(26/32)	[19]
*Agrostis scabra/Agrostis stolonifera*	root	nd; Penncross	20 °C, 30 °C, 40 °C; 2 d, 10 d	2DE, MALDI-TOF/TOF MS	70	67(23/44)	[20]
*Agrostis scabra/Agrostis stolonifera*	leaf	nd; Penncross	40 °C/35 °C day/night; 2 d, 10 d	2DE-DIGE	71	71(57/14)	[21]
*Arabidopsis thaliana*	leaf	Columbia (Col-0)	40 °C; 6 h	2DE, MALDI-TOF MS, ESI-Q-Trap MS	37	33(12/21)	[22]
*Avena sativa*	seed	nd	35 °C, 45 °C, 50 °C; 24 h, 2 d	2DE, ESI-Orbitrap MS	21	21(2/19)	[23]
*Brassica oleracea*	leaf	TSS-AVRDC-2; B-75	40 °C; 3 d	2DE, MALDI-TOF MS	24	24(10/13/1)	[24]
*Carissa spinarum*	leaf	nd	42 °C/35 °C day/night; 48 h, 120 h	2DE, MALDI-TOF/TOF MS	49	26(13/13)	[25]
*Glycine max*	leaf, stem, root	Enrei	40 °C; 6, 12, 24 h	2DE, MALDI-TOF MSESI-Orbitrap MS	150	150(122/28)	[26]
*Glycine max*	leaf	Surge; Davison	40 °C/35 °C day/night; 6 d	2DE-DIGE, MALDI-TOF MS	88	44(19/25)	[27]
*Glycine max*	seed	Ningzhen No. 1	40 °C/30 °C day/night; 24, 96, 168 h	2DE, MALDI-TOF MS	42	42(22/20)	[28]
*Hordeum spontaneum*	leaf	nd	42 °C, 2 h	2DE-DIGE, MALDI-TOF/TOF MS	20	20(12/8)	[29]
*Hordeum vulgare*	leaf	Arta; Keel	36 °C/32 °C day/night; 7 d	2DE, MALDI-TOF/TOF MS	99	99(67/32)	[30]
*Medicago sativa*	leaf	Huaiyin	36 °C; 24, 48, 72 h	2DE, MALDI-TOF/TOF MS	81	81	[31]
*Miscanthus sinensis*	leaf	Kosung	42 °C; 24, 48 h	2DE, MALDI-TOF MS, 2DE, MALDI-TOF/TOF MS	55	55(30/25)	[32]
*Oryza meridionalis*	leaf	nd	45 °C; 24 h	2DE, ESI-Q-Trap MS	50	38(22/16)	[33]
*Oryza sativa*	leaf	Dongjin	40 °C; 12, 24 h	2DE, MALDI-TOF MS	73	56(47/9)	[34]
*Oryza sativa*	leaf	nd	35, 40, 45 °C; 48 h	2DE, MALDI-TOF MS	63	52(28/24)	[35]
*Oryza sativa*	leaf	N22	42 °C/32 °C day/night; 24 h	2DE, MALDI-TOF MS	111	52(37/15)	[36]
*Oryza sativa*	leaf, spikelet	N22; Gharib	28 °C; 12 h	2DE, MALDI-TOF MS	36	36	[37]
*Oryza sativa*	cell suspension cultures	Doongara	44 °C; 3 d	1DE, ESI-Q-Trap MS	139	139	[38]
*Oryza sativa*	grain	Khao Dawk Mali 105	40 °C; 3 d	ESI-Orbitap MS	822	822	[39]
*Oryza sativa*	leaf	Nipponbare	42 °C, 12 h, 24 h	2DE, MALDI-TOF/TOF MS	12	12(9/3)	[40] *
*Pinellia ternata*	leaf	nd	38 °C; 24 h	2DE, MALDI-TOF/TOF MS	27	24(17/7)	[41]
*Portulaca oleracea*	leaf	nd	35 °C; 6h, 12 h, 24 h	2D, MALDI-TOF/TOF MS	154	51(36/15)	[42]
*Prunus persica*	mesocarp	nd	39 °C; 3 d	2DE, MALDI-TOF/TOF MS	44	33(15/18)	[43]
*Raphanus sativus*	leaf	NAU-08Hr-10	40 °C; 0 h, 12 h, 24 h	2DE, MALDI-TOF/TOF MS	11	11(4/1/6)	[44]
*Triticum aestivum*	endosperm	Récital	34 °C/10 °C day/night	2DE, MALDI-TOF MS	37	23(22/1)	[45]
*Triticum aestivum*	non-prolamins	Thésée	34 °C/10 °C day/night	2DE, MALDI-TOF MS	42	24(16/8)	[46]
*Triticum aestivum*	seed	Svevo	37 °C/17 °C day/night; 5 d	2DE, MALDI-TOF/TOF MS	47	47(37/10)	[47]
*Triticum aestivum*	spikelet	Vinjett	32 °C/24 °C day/night; 10 d	2DE, MALDI-TOF/TOF MS	57	57(36/21)	[48]
*Triticum aestivum*	leaf	810; 1039	35 °C/26 °C day/night; 5 d	2DE, MALDI-TOF MS	49	49(32/11, 12/21)	[49]
*Vitis vinifera*	leaf	Cabernet Sauvignon	43 °C; 6 h	iTRAQ, ESI-Q-TOF MS	113	113(48/65)	[50]
*Zea mays*	leaf	Zhengdan 958	heat from 28 to 42 °C, total 8 h	iTRAQ, ESI-Orbitrap MS	172	172(77/95)	[51] *

^(a)^ The number of identified protein identities included all the protein isoforms generated from gene variable splicing and post-translational modifications in these original publications. ^(b)^ The number of non-redundant protein identities whose members have similar protein sequence homology and functional domain (increased protein number/decreased protein number). The references labeled with * are phosphoproteomic studies. The information of these heat-responsive proteins is listed in Tables S1 and S2. 2DE, two-dimensional electrophoretic; d, days; DIGE, two-dimensional fluorescence difference in gel; ESI-Q-TOF MS, electrospray ionization quadrupole time-of-flight mass; iTRAQ, isobaric tags for relative and absolute quantitation; MALDI-TOF MS, matrix-assisted laser desorption/ionization time-of-flight mass; MALDI-TOF/TOF MS, matrix-assisted laser desorption/ionization tandem time-of-flight mass; nd, not detected.

**Table 2:** Heat stress-responsive proteins involved in photosynthesis, carbon metabolism and signaling identified in leaves by proteomics studies.

Protein Name	Abbreviation	Plant Species
**1. Photoreaction**		
Magnesium chelatase subunit	CHLI	Po; Gm; Vv
Chlorophyll a/b binding protein	LHC	Ta; Pt; Os; Vv; MS
Oxygen-evolving enhancer protein 1	OEE1	Gm; Aa; Ta; Os; Cs; Asc; Ast; At
Oxygen-evolving enhancer protein 2	OEE2	Asc; Rs; Gm; Os; Gm; At; Ms
Photosystem I PsaA subunit	PsaA	Aa; Vv
Photosystem I PsaB subunit	PsaB	Aa
Photosystem I PsaD subunit	PsaD	Aa; At; Vv; Ms
Photosystem I PsaE subunit	PsaE	Asc; Ast
Photosystem I PsaN subunit	PsaN	Aa; Asc; Vv
Photosystem I PsaL subunit	PsaL	Vv
Photosystem I PsaF subunit	PsaF	Vv
Photosystem I PsaH subunit	PsaH	Vv
Photosystem II PsbA subunit	PsbA	Vv
Photosystem II PsbP subunit	PsbP	Os
Photosystem II PsbO subunit	PsbO	Os
Photosystem II PsbS subunit	PsbS	Vv
Photosystem II PsbR subunit	PsbR	Vv
**2. Calvin cycle**		
RuBisCO activase	RCA	Os; Ta; Om; Asc; Ast; Os; At; Vv; Ms
RuBisCO large subunit	Rubisco LS	Gm; Os; Bo; Aa; Ta; Po; Cs; Cs; Om; Asc; Ast; At; Vv; Ms
RuBisCO small subunit	Rubisco SS	Ta; Gm; Os; Bo; Asc; Ast; At; Vv; Ms
Phosphoribulokinase	PRK	Os; Om; Asc; Ast; Vv
Transketolase	TK	Os; Om
Sedoheptulose-1,7-bisphosphatase	SBPase	Gm; Ta; Om
Chloroplast fructose-bisphosphate aldolase	FBPA	At; Asc; Ast; Gm; Ta; Vv
C4-specific pyruvate orthophosphate dikinase	PPDK	Ta
Carbonic anhydrase	CA	Asc; Ast; Gm; Vv
Phosphoglycerate kinase	PGK	Om
Glyceraldehyde-3-phosphate dehydrogenase	GAPDH	At; Ast; Asc; Ta; Ms Vv,
**3. Photorespiration**		
Glycolate oxidase	GO	Po
Glycine dehydrogenase	GLDC	Os; Om
Glycine decarboxylase	GDC	Ta; Os
Glutamine synthetase	GS	Os
**4. ATP synthesis and electrical transport chain**		
ATP synthase CF (0) b subunit	CF0	Vv
ADP, ATP carrier protein 1	AAC	Aa
ATP synthase α subunit	α	Os; Ta
ATP synthase β subunit	β	Aa; Asc; Ast; Bo; Gm; Om; Os; Ta; Vv
ATP synthase γ subunit	γ	Asc; Ast; At; Vv
ATP synthase δ subunit	δ	Cs; Vv
Cytochrome c oxidase assembly protein	COX	Po
Rieske Fe/S protein of cytochrome b6/f complex	Fe/S	Pt
Ferredoxin-NADP(H) oxidoreductase	FNR	Om; Gm; Vv
Plastocyanin	PC	Ms
**5. Glycolysis**		
Triosephosphate isomerase	TPI	Gm; Ta; Cs; Os; At
Alcohol dehydrogenase	ADH	Gm; Po
Fructokinase	FK	Gm
Fructose-bisphosphate aldolase	FBPA	Gm; Asc; Ast; Os
Glyceraldehyde-3-phosphate dehydrogenase	GAPDH	Gm; Gm; Ta; Asc; Ast; Os; At
Phosphoglycerate kinase	PGK	Gm; Asc; Ast; Os
Phosphoglycerate mutase	PGM	Ta
Pyruvate kinase	PK	Ast
Enolase		Ta; Asc; Ast; Ms
**6. Pentose phosphate pathway**		
Phosphogluconate dehydrogenase	PGD	Asc; Ast; Vv
Transketolase	TK	Os
**7. TCA cycle**		
Malate dehydrogenase	MDH	Os; Gm; Asc; Ast; Vv; Ms
Isocitrate dehydrogenase	IDH	Ta
Cytoplasmic aconitate hydratase	AH	Asc; Ast
Phosphoenolpyruvate carboxylase	PEPC	Po; Gm; Aa
Dihydrolipoyl dehydrogenase	DLDH	Ta
Pyruvate dehydrogenase	PDH	Os; Cs; Gm
Citrate synthase	CS	Ms; Vv
Fumarate hydratase	FH	Ms
**8. Signaling**		
Phospholipase C	PLC	Ta
GTP-binding protein	G protein	Po
Ras-related nuclear protein 1A	Ran 1A	Asc; Ast
Ras-related protein	Rab	Gm
Ca2+-transporting ATPase-like protein		Aa
Calcium/calmodulin-dependent protein kinase	CDPK	Gm
Calcium-binding protein	CaB	Aa
BRI1-KD interacting protein 114		Ta
Nucleoside diphosphate kinase	NDPK	As; Gm; Ms; Os
WRKY transcriptional factor	WRKY	Po
MYB family transcription factor	MYB	Po

Aa, *Agave americana*; Asc, *Agrostis scabra*; Ast, *Agrostis stolonifera*; At, *Arabidopsis thaliana*; Cs, *Carissa spinarum*; Gm, *Glycine max*; Ms, *Medicago sativa*; Om, *Oryza meridionalis*; Os, *Oryza sativa*; Pt, *Pinellia ternata*; Po, *Portulaca oleracea*; Ta, *Triticum aestivum*. Vv, *Vitis vinifera*. Please refer to Table S1 for details.

## Supplementary Materials

Supplementary materials can be found at www.mdpi.com/1422-0067/18/10/2191/s1.

## Abbreviations

2DE	Two-dimensional electrophoretic
BRI1-KD	Brassinosteroid-insensitive I-kinase domain
C_4_-PPDK	C_4_-specific pyruvate orthophosphate dikinase
CA	Carbonic anhydrase
CDPK	Calcium-dependent protein kinases
Chl	Chlorophyll
DIGE	Two-dimensional fluorescence difference in gel
ESI-Q-TOF	Electrospray ionization quadrupole time-of-flight
fstH	Filamentation temperature-sensitive H
FBPA	Fructose-bisphosphate aldolase
G protein	GTP-binding protein
GAPDH	Glyceraldehyde 3-phosphate dehydrogenase
GDC	Glycine decarboxylase
GLDC	Glycine dehydrogenases
GO	Glycolate oxidase
HSF	Heat shock factor
HSPs	Heat shock proteins
iTRAQ	Isobaric tags for relative and absolute quantitation
LHC	Light-harvesting chlorophyll a/b binding protein
MALDI	Matrix-assisted laser desorption/ionization
MAPK	Mitogen-activated protein kinase
MAPK	Mitogen-activated protein kinase
NDPK	Nucleoside diphosphate kinase
OEC	Oxygen-evolving complex
PEPCK	Phosphoenolpyruvate carboxykinase
PLC	Phospholipase C
POR	Protochlorophyllide reductase
PS	Photosystem
ROS	Reactive oxygen species
RCA	Rubisco activase
RuBisCO	Ribulose-1,5-bisphosphate carboxylase/oxygenase
RuBP	Ribulose bisphosphate
SBPase	Sedoheptulose-1,7-bisphosphatase
TK	Chloroplast transketolase
TOF	Time-of-flight
TOF/TOF	Tandem time-of-flight
WOC	Water oxidizing complex
